# Olivetolic acid, a cannabinoid precursor in *Cannabis sativa*, but not CBGA methyl ester exhibits a modest anticonvulsant effect in a mouse model of Dravet syndrome

**DOI:** 10.1186/s42238-021-00113-w

**Published:** 2022-01-04

**Authors:** Lyndsey L. Anderson, Michael Udoh, Declan Everett-Morgan, Marika Heblinski, Iain S. McGregor, Samuel D. Banister, Jonathon C. Arnold

**Affiliations:** 1 Brain and Mind Centre, The University of Sydney, Sydney, NSW 2050 Australia; 2 Discipline of Pharmacology, School of Pharmacy, Faculty of Medicine and Health, The University of Sydney, Sydney, NSW 2006 Australia; 3 Lambert Initiative for Cannabinoid Therapeutics, The University of Sydney, Sydney, NSW 2050 Australia; 4 School of Psychology, Faculty of Science, The University of Sydney, Sydney, NSW 2006 Australia; 5 School of Chemistry, Faculty of Science, The University of Sydney, Sydney, NSW 2006 Australia

**Keywords:** Cannabis, Olivetolic acid, CBGA, Dravet syndrome, Epilepsy, Anticonvulsant

## Abstract

**Objective:**

Cannabigerolic acid (CBGA), a precursor cannabinoid in *Cannabis sativa*, has recently been found to have anticonvulsant properties in the *Scn1a*^+/-^ mouse model of Dravet syndrome. Poor brain penetration and chemical instability of CBGA limits its potential as an anticonvulsant therapy. Here, we examined whether CBGA methyl ester, a more stable analogue of CBGA, might have superior pharmacokinetic and anticonvulsant properties. In addition, we examined whether olivetolic acid, the biosynthetic precursor to CBGA with a truncated (des-geranyl) form, might possess minimum structural requirements for anticonvulsant activity. We also examined whether olivetolic acid and CBGA methyl ester retain activity at the epilepsy-relevant drug targets of CBGA: G-protein-coupled receptor 55 (GPR55) and T-type calcium channels.

**Methods:**

The brain and plasma pharmacokinetic profiles of CBGA methyl ester and olivetolic acid were examined following 10 mg/kg intraperitoneal (i.p.) administration in mice (*n* = 4). The anticonvulsant potential of each was examined in male and female *Scn1a*^+/-^ mice (*n* = 17–19) against hyperthermia-induced seizures (10–100 mg/kg, i.p.). CBGA methyl ester and olivetolic acid were also screened in vitro against T-type calcium channels and GPR55 using intracellular calcium and ERK phosphorylation assays, respectively.

**Results:**

CBGA methyl ester exhibited relatively limited brain penetration (13%), although somewhat superior to that of 2% for CBGA. No anticonvulsant effects were observed against thermally induced seizures in *Scn1a*^+/-^ mice. Olivetolic acid also showed poor brain penetration (1%) but had a modest anticonvulsant effect in *Scn1a*^+/-^ mice increasing the thermally induced seizure temperature threshold by approximately 0.4°C at a dose of 100 mg/kg. Neither CBGA methyl ester nor olivetolic acid displayed pharmacological activity at GPR55 or T-type calcium channels.

**Conclusions:**

Olivetolic acid displayed modest anticonvulsant activity against hyperthermia-induced seizures in the *Scn1a*^+/-^ mouse model of Dravet syndrome despite poor brain penetration. The effect was, however, comparable to the known anticonvulsant cannabinoid cannabidiol in this model. Future studies could explore the anticonvulsant mechanism(s) of action of olivetolic acid and examine whether its anticonvulsant effect extends to other seizure types.

**Supplementary Information:**

The online version contains supplementary material available at 10.1186/s42238-021-00113-w.

## Introduction

The anticonvulsant properties of phytocannabinoids are now broadly acknowledged, with cannabidiol (CBD), a non-intoxicating component of *Cannabis sativa*, recently approved in many countries for the treatment of intractable childhood epilepsies such as Dravet syndrome and Lennox-Gastaut syndrome. This emerging clinical use of CBD as an effective anticonvulsant has prompted the question of whether other phytocannabinoids may have even greater potential as anticonvulsants.

More than 140 phytocannabinoids have been identified in *Cannabis sativa* and recent preclinical research shows a growing number with anticonvulsant properties across a variety of animal models of epilepsy (Anderson et al., 2019b, 2020, 2021a, b; Benson et al. 2020; Chiu et al. 1979; Davis and Hatoum 1983; Hill et al. 2010, 2012; Kaplan et al. 2017; Karler and Turkanis 1979). This includes phytocannabinoid acids that are synthesized enzymatically in the cannabis plant, as well as neutral phytocannabinoids that are formed from the non-enzymatic decarboxylation of phytocannabinoid acids via thermal degradation.

Cannabigerolic acid (CBGA), formed by the geranylation of olivetolic acid, is a phytocannabinoid acid and the primary precursor to phytocannabinoids in *Cannabis sativa.* Enzymatic processes convert CBGA to cannabidiolic acid (CBDA) and Δ^9^-tetrahydrocannabinolic acid (Δ^9^-THCA), which then decarboxylate to form the highly prevalent and widely consumed phytocannabinoids CBD and Δ^9^-THC.

The pharmacological and potential therapeutic actions of CBGA are largely unknown, although a recent study from our laboratory demonstrated that CBGA has anticonvulsant properties in both the *Scn1a*^+/-^ mouse model of Dravet syndrome and in the maximal electroshock (MES) seizure threshold test (Anderson et al. 2021b). The potential of CBGA as an anticonvulsant appears limited due to its poor brain uptake (~2%), likely due to its carboxylic acid moiety, lipophilicity, and rotatable bond count (Anderson et al. 2019b; Rankovic 2015a, 2015b). Only 3–4% of the central nervous system (CNS) drugs are estimated to feature a carboxylic acid group, with exceedingly few single carboxylic acid-containing CNS drugs having an acid-base dissociation constant (p*K*_a_) below 6 (Ghose et al. 2012; Manallack 2007).

CBGA is predicted to have a strongest acidic pKa value of 2.92 (ChemAxon; Budapest, HUN). Moreover, the chemical instability of CBGA renders it a poor candidate for further conventional development due to the lability of its carboxylic acid moiety and facile, degradative conversion to cannabigerol (CBG) under ambient conditions.

**Fig. 1:**
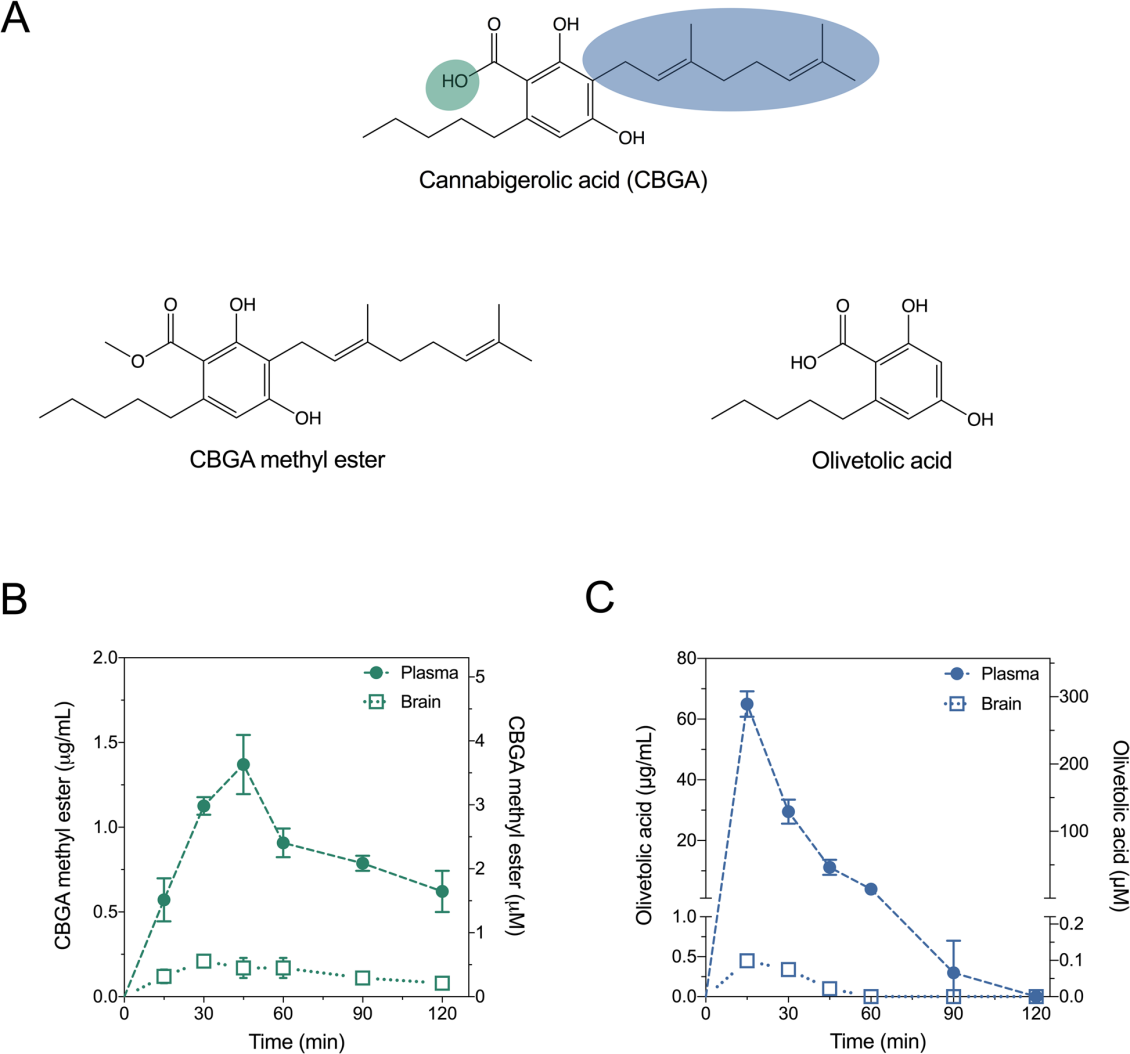
Pharmacokinetic analysis of CBGA methyl ester and olivetolic acid in the mouse plasma and brain samples. **A** Comparison of the chemical structure of cannabigerolic acid (CBGA) to those of CBGA methyl ester and olivetolic acid. Concentration-time curves for **B** CBGA methyl ester in the plasma and brain and **C** olivetolic acid in the plasma and brain following 10 mg/kg i.p. injections. Concentrations are depicted as both mass concentrations (left *y*-axis) and molar concentrations (right *y*-axis). Data are expressed as means ± SEM, with *n* = 4 per time point

A potential strategy to mitigate facile decarboxylation of CBGA and improve its brain penetration is esterification of the ionisable carboxylic acid group to give the non-ionisable CBGA methyl ester (Fig. 1A). Analogous esterification of cannabidiolic acid (CBDA) to give CBDA methyl ester (HU-580) was reported to improve stability and in vivo efficacy as an anti-emetic and anxiolytic (Pertwee et al. 2018). Therefore, it is of interest to determine whether the pharmacological activity and anticonvulsant profile CBGA are retained, or even enhanced, with CBGA methyl ester.

The present study also sought to extend on the prior findings with CBGA by exploring structure-activity relationships around this natural product; specifically, minimum structural requirements for retention of anticonvulsant activity. Geranylpyrophosphate: olivetolate geranyltransferase (CBGA synthase) catalyzes the geranylation of olivetolic acid to yield CBGA (Fellermeier and Zenk 1998). The importance of the geranyl chain to the anticonvulsant efficacy and pharmacological actions of CBGA is unknown, and truncation of this group (to give olivetolic acid) reduces lipophilicity and rotatable bond count, leading to a physicochemical profile more favorably concordant with CNS drugs (Fig. 1A).

The present study assessed the anticonvulsant potential of the CBGA-related compounds, CBGA methyl ester and olivetolic acid, against hyperthermia-induced seizures in the *Scn1a*^+/-^ mouse model of Dravet syndrome. The majority of Dravet syndrome patients have a loss-of-function mutation in *SCN1A*, the gene that encodes the voltage-gated sodium channel Na_v_1.1 (Meisler et al. 2010; Yamakawa 2009). Heterozygous deletion of *Scn1a* (*Scn1a*^+/-^) in mice replicates the phenotypes of Dravet syndrome patients, including susceptibility to hyperthermia-induced seizures (Ito et al. 2013; Miller et al. 2014; Yu et al. 2006). We also conducted a preliminary characterization of the pharmacological action of CBGA methyl ester and olivetolic acid at known molecular targets of CBGA and other anticonvulsant phytocannabinoids, namely T-type calcium channels and G-protein-coupled receptor 55 (GPR55) (Anavi-Goffer et al. 2012; Anderson et al. 2021a, 2021b; Bladen et al. 2021; Sylantyev et al. 2013).

## Materials and methods

### Compounds

CBGA methyl ester was synthesized by Acme Bioscience, Inc (Palo Alto, USA), with > 98% purity. Olivetolic acid was purchased from Toronto Research Chemicals Inc. (North York, CAN), with >97% purity. Compounds were prepared fresh on the day of the experiment as solutions in ethanol:Tween80:saline (1:1:18) and administered as an intraperitoneal (i.p.) injection in a volume of 10 mL/kg. CID16020046 and NNC 55-0396 were purchased from Tocris Biosciences (Bristol, GBR) and Cayman Chemical (Ann Arbor, USA), respectively. Lysophosphatidylinositol (LPI) was purchased from Sigma-Aldrich (St. Louis, USA).

### Animals

All animal care and procedures were approved by the University of Sydney Animal Ethics Committee in accordance with the Australian Code of Practice for the Care and Use of Animals for Scientific Purposes. Mice were group-housed in specific pathogen-free mouse facilities under standard conditions (12 h light/12 h dark cycle) with ad libitum access to food and water. Mice heterozygous for *Scn1a* (*Scn1a*^+/-^) were purchased from The Jackson Laboratory (stock 37107-JAX; Bar Harbor, USA) and maintained as a congenic line on the 129S6/SvEvTac background (129.*Scn1a*^+/-^). 129.*Scn1a*^+/-^ mice were bred with C57BL/6J mice to generate experimental mice. The *Scn1a* genotype was determined as previously described (Miller et al. 2014).

### Pharmacokinetic analysis

Wildtype mice (postnatal day 21-28, P21-28) received an i.p. injection of 10 mg/kg CBGA methyl ester or olivetolic acid. At selected time points (15–120 min) mice were anesthetized with isoflurane, whole blood was collected by cardiac puncture and brains were harvested. The plasma was isolated by centrifugation and samples were stored at −80°C until assayed. Concentrations in the plasma and brain samples at each time point were averaged, and pharmacokinetic parameters were calculated by noncompartmental analysis as previously described (Hawkins et al. 2017).

### Analytical analysis

The plasma and brain samples were prepared for LC-MS/MS analysis as previously described (Anderson et al. 2019b, 2020). Briefly, acetonitrile was used to precipitate proteins and extract CBGA methyl ester and olivetolic acid from the biological matrices. The brain samples were additionally filtered through Amicon Ultracel 3K (Merck-Millipore; Burlington, USA) filtration devices. The plasma and brain samples underwent supported-liquid extraction with methyl *tert-*butyl ether using Biotage Isolute SLE+ columns (Uppsala, SWE) and were then evaporated to dryness with N_2_. Residues were reconstituted in actetonitrile and 0.1% formic acid in water (1:1, v/v) for analysis using a Shimadzu Nexera ultra-HPLC coupled to a Shimadzu 8030 triple quadrupole mass spectrometer (Shimadzu Corp.; Kyoto, JPN). The mass spectrometer was operated in positive electrospray ionization mode for CBGA methyl ester and negative electrospray ionization mode for olivetolic acid with multiple reaction monitoring and the following mass transition pairs (*m/z*): 375.20 > 343.25, 375.20 > 219.10 and 375.20 > 251.15 (CBGA-methylester) and 223.25 > 179.25 (olivetolic acid). Quantification of analytes in the plasma and brain samples was achieved by comparing samples to standards prepared with known amounts of compound.

### Hyperthermia-induced seizures

Hyperthermia-induced seizure experiments were conducted on male and female *Scn1a*^+/-^ mice at P14-16 as previously described (Hawkins et al. 2017). No significant sex differences were observed so males and females were combined across groups. Briefly, for CBGA methyl ester experimental animals a RET-3 rectal temperature probe was inserted 30 min after i.p. injections of vehicle or CBGA methyl ester. Mice acclimated to the probe for 5 min before hyperthermia protocol began. Olivetolic acid experimental animals acclimated to the rectal temperature probe for 5 min then received an i.p. injection of vehicle or olivetolic acid immediately before the hyperthermia protocol was initiated. Mouse core body temperature was elevated 0.5°C every 2 min until the onset of first clonic convulsion with loss of posture or until 42.5°C was reached. The dose range (10, 30, or 100 mg/kg) was chosen to provide a point of direct comparison to CBGA, which is anticonvulsant at 30 and 100 mg/kg. Seizure threshold temperatures were compared in GraphPad Prism 8.2 (LaJolla, USA) using Mantel-Cox logrank test and *p* < 0.05 was considered statistically significant. Following the hyperthermia-induced seizure protocol, the plasma samples were isolated as described for the pharmacokinetic analysis from a cohort of experimental mice.

### Gpr55 pERK assay

ERK phosphorylation (pERK) was detected using the AlphaScreen® SureFire® Phospho-ERK1/2 assay (Perkin Elmer; Waltham, USA) as previously described (Anderson et al. 2021b). Briefly, the human embryonic kidney (HEK) 293 cells stably expressing human GPR55 in L-15 media were plated (110,000/well) into a Corning CellBIND (Corning Inc; Corning, USA) clear, flat-bottomed 96-well plate and incubated 20–24 h at 37°C in ambient CO_2_. Media was aspirated, 50 μL phenol red-free DMEM/F-12 was added to each well and incubated for 2–3 h at 37°C in a humidified 5% CO_2_ atmosphere. Compounds (10 μM in phenol red-free DMEM/F-12) were added and incubated for 20 min before media was aspirated and lysis buffer was added. Lysates (5 μL) were incubated with reaction mixture (7 μL) for 3.5 h at room temperature. A CLARIOstar plate reader (BMG Labtech; Offenburg, GER) was used to take fluorescence readings over 740 msec (excitation 680 nm, emission 520–620 nm). Fluorescence readings were normalized to those of 4 μM LPI, the endogenous GPR55 agonist. No effect of LPI (10 μM) is observed in control HEK293 cells (Supplemental Fig. 1). Final DMSO concentrations were ≤ 0.2%. Statistical comparisons were made in GraphPad Prism using one-way ANOVA followed by Dunnett’s post hoc and *p* < 0.05 was considered statistically significant.

### Measurement of intracellular calcium

Changes in intracellular calcium concentrations were measured in Flp-In™ T-REx™ HEK stably expressing either Ca_V_3.1, Ca_V_3.2, or Ca_V_3.3 using Calcium 5 dye (Molecular Devices; Sunnyvale, USA) as previously described (Bladen et al. 2021). Briefly, cells (120,000/well) were plated into a black, clear-bottomed poly-d-lysine coated, 96-well plate in the presence of 2 μg/mL tetracycline to induce channel expression and incubated overnight at 37°C in ambient CO_2_. Calcium 5 dye was diluted in HEPES-buffered low potassium Hanks Balanced Salt Solution (HBSS) at 50% of manufacturer’s recommendation. Dye solution containing probenecid (2.5 mM) was added and incubated for 60 min at 37°C in ambient CO_2_. Compounds were prepared as stock solutions in DMSO and diluted in HBSS with 0.01% bovine serum albumin. Compounds were applied for 5 min, then 10 mM CaCl_2_ was added. A FlexStation 3 Microplate reader (Molecular Devices; San Jose, USA) was used to take fluorescence readings every 2 s (excitation 485 nm, emission 525 nm). Baseline recordings were taken as the average fluorescence over 20 s preceding CaCl_2_ addition. Changes in fluorescence were measured as the maximal increase in fluorescence from baseline following CaCl_2_ addition and normalized to the response of vehicle. CaCl_2_ addition produced no effect in non-induced cells (Supplemental Fig. 2). Final drug concentrations had ≤ 0.1% DMSO. Statistical comparisons were made in GraphPad Prism using one-way ANOVA followed by Dunnett’s post hoc, and *p* < 0.05 was considered statistically significant.

## Results

### Pharmacokinetic profiles of CBGA methyl ester and olivetolic acid in the mouse plasma and brain

In order to inform subsequent in vivo experiments, pharmacokinetic parameters were first calculated for CBGA methyl ester and olivetolic acid in mouse plasma and brain following 10 mg/kg intraperitoneal (i.p.) administration. Absorption of CBGA methyl ester into the plasma was relatively slow with a t_max_ of 45 min (Fig. 1B, Table 1). Elimination of CBGA methyl ester was also relatively slow with plasma and brain t_1/2_ values of 81 min and 62 min, respectively. Overall drug exposure of CBGA methyl ester determined by AUC values was lower in the brain than it was in the plasma (brain-plasma ratio 0.13).

**Table 1:** Pharmacokinetic parameters in mouse plasma and brain

	CBGA methyl ester		Olivetolic acid	
	(10 mg/kg, i.p.)		(10 mg/kg, i.p.)	
	Plasma	Brain	Plasma	Brain
C_max_ (μg/mL)	1.3 ± 0.3	0.21 ± 0.03*	65 ± 4	0.45 ± 0.06*
t_max_ (min)	45	30	15	15
t_1/2_ (min)	81	62	10	14
AUC (μg•min/mL)	171	22	1105	13
Brain-plasma ratio	0.13		0.01	

*Converted from measured concentrations (ng/mg brain) assuming density of 1 g/mL

Olivetolic acid was rapidly absorbed in the plasma (t_max_ 15 min) and achieved a high maximal plasma concentration (C_max_ 65 ± 4 μg/mL; Fig. 1C, Table 1). Distribution into the brain tissue was also rapid (t_max_ 15 min); however, olivetolic acid concentrations in the brain were low (C_max_ 0.45 ± 0.06 ng/mg brain). Olivetolic acid was rapidly eliminated from both plasma and brain with half-lives less than 15 min. Olivetolic acid exhibited poor brain penetration with a brain-plasma ratio of 0.01.

### Olivetolic acid is anticonvulsant against hyperthermia-induced seizures in *Scn1a*^+/-^ mice

**Fig. 2:**
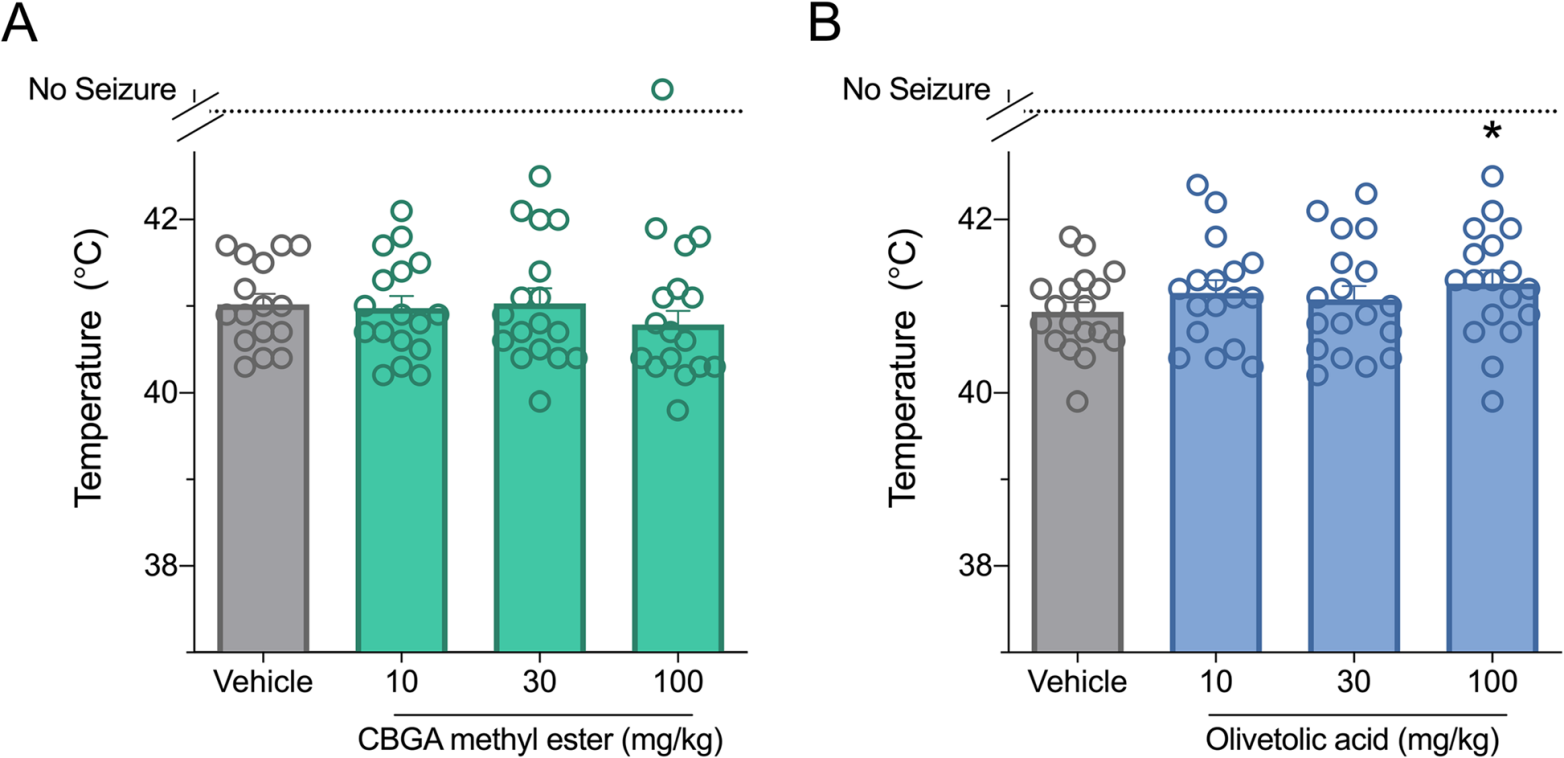
Olivetolic acid is anticonvulsant against hyperthermia-induced seizures in *Scn1a*^+/-^ mice. Threshold temperature of individual mice for generalized tonic-clonic seizure (GTCS) induced by hyperthermia in male and female *Scn1a*^+/-^ mice following acute intraperitoneal treatment with varying doses of **A** CBGA methyl ester or **B** olivetolic acid. Olivetolic acid (100 mg/kg) significantly increased the temperature threshold for GTCS, indicating an anticonvulsant effect. CBGA methyl ester had no effect on thermally induced seizures. The average temperatures of seizure induction are depicted by the bars and error bars represent SEM, with *n* = 17–19 per group (*p < 0.05, Mantel-Cox logrank)

Next, we evaluated the efficacy of CBGA methyl ester and olivetolic acid against hyperthermia-induced seizures in *Scn1a*^+/-^ mice, which models febrile seizures that occur in children with Dravet syndrome. Between P14 and P16, *Scn1a*^+/-^ mice were treated with a single i.p. injection of vehicle, CBGA methyl ester or olivetolic acid and challenged with a thermal event. CBGA methyl ester had no effect on hyperthermia-induced seizures at any dose tested (Fig. 2A). Olivetolic acid, however, showed a modest but significant anticonvulsant effect at a dose of 100 mg/kg (Fig. 2B) with a significant increase in generalized tonic-clonic seizure (GTCS) temperature threshold (*p* = 0.0331). Plasma concentrations of CBGA methyl ester and olivetolic acid achieved in experimental animals are presented in Table 2.

**Table 2:** Plasma concentrations of compounds in experimental *Scn1a*^+-^ mice

Compound	Dose (mg/kg)	Plasma concentration (μg/mL)
CBGA methyl ester	10	837±212 ng/mL
	30	4.7±0.6
	100	6.2±0.6
Olivetolic acid	10	65±5
	30	89±12
	100	116±27

### Pharmacological characterization at GPR55 and T-type calcium channels

**Fig. 3:**
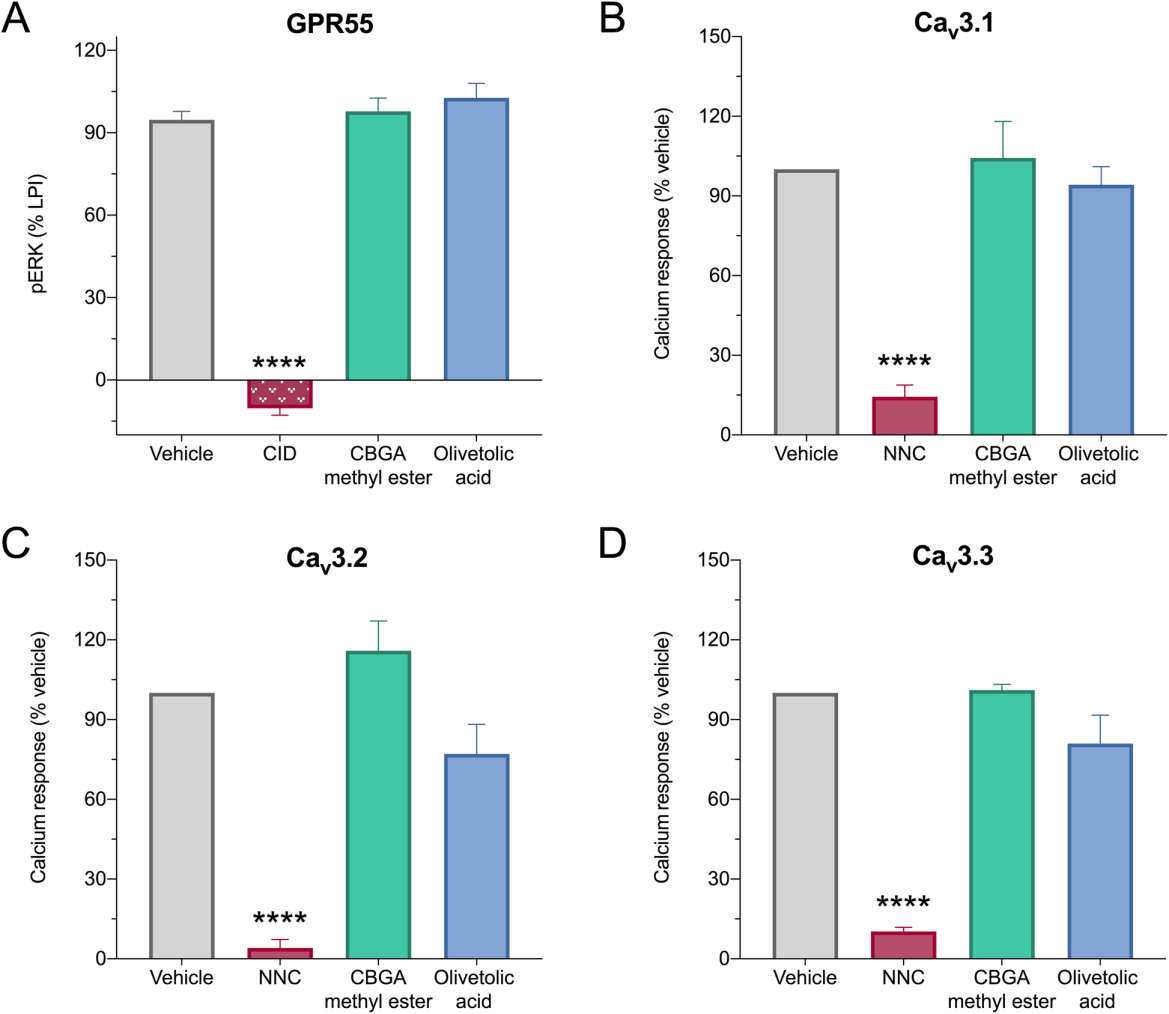
CBGA methyl ester and olivetolic acid do not inhibit GPR55 or T-type calcium channels. **A** HEK293 cells expressing human GPR55 were used to screen compounds for antagonist activity. ERK phosphorylation (pERK) following treatment with EC_80_ concentrations of the endogenous ligand LPI was determined by Alphascreen Phospho-ERK1/2 assay and used as a measure of GPR55 activation. Compounds were screened at a concentration of 10 μM (CID, CID16020046). Data are expressed as a mean ± SEM, with *n* = 5 per group (****p < 0.0001, one-way ANOVA followed by Dunnett’s post hoc). HEK293 cells expressing human **B** Ca_v_3.1, **C** Ca_v_3.2, or **D** Ca_v_3.3 were used to screen compounds for antagonist activity. Changes in intracellular calcium concentrations were used as a measure of calcium channel activity. Compounds were screened at a concentration of 10 μM (NNC, NNC 55-0396). Data are expressed as a mean ± SEM, with *n* = 5 per group (****p < 0.0001, one-way ANOVA followed by Dunnett’s post hoc)

The pharmacological actions of CBGA methyl ester and olivetolic acid were examined at two epilepsy-relevant targets of CBGA: GPR55 and T-type calcium channels (Anderson et al. 2021b and unpublished data). First, the inhibitory potential of these compounds were screened at GPR55. The endogenous ligand LPI was applied at an EC_80_ concentration to HEK293 cells stably expressing human GPR55 and ERK phosphorylation was measured (Anderson et al. 2021b). CBGA methyl ester and olivetolic acid were screened at 10 μM and GPR55 activity was calculated relative to agonist alone (Fig. 3A). CBGA methyl ester and olivetolic acid did not inhibit GPR55 activity. GPR55 activity was completely inhibited by the positive control inhibitor, CID16020046 (CID).

Next, we screened the inhibitory potential of CBGA methyl ester and olivetolic acid in vitro at the three T-type calcium channel isoforms: Ca_V_3.1, Ca_V_3.2, and Ca_V_3.3. HEK293 cells expressing the human variants of each isoform were used. CBGA methyl ester and olivetolic acid were screened at 10 μM, and change in intracellular calcium concentration was calculated relative to vehicle. Neither CBGA methyl ester nor olivetolic acid had any effect on the activity of these channels (Fig. 3B–D). As expected, the positive control inhibitor NNC 55-0396 (NNC) significantly inhibited all three T-type calcium channel isoforms.

## Discussion

CBGA has anticonvulsant effects against hyperthermia-induced seizures in the *Scn1a*^+/-^ mouse model of Dravet syndrome. Unfortunately, CBGA has less than optimal pharmacokinetic and physicochemical properties and exhibited divergent effects on seizures across multiple preclinical epilepsy models (Anderson et al. 2021b). We, therefore, aimed to determine whether the structurally related compounds CBGA methyl ester and olivetolic acid had superior anticonvulsant properties and more favorable pharmacokinetic properties.

Our approach was inspired by reports of HU-580, a stable CBDA analog obtained via substitution of the carboxylic acid (COOH) moiety with a methyl ester (COOCH_3_). Pertwee et al. (2018) demonstrated improved stability of HU-580, greater potency at 5HT_1A_ receptors and increased anti-nausea and anti-anxiety effects in rats relative to the CBDA parent (Pertwee et al. 2018). The improved physiochemical properties of CBGA methyl ester suggest that it would have better brain penetration and, as a result, enhanced anticonvulsant efficacy compared to CBGA. Here, we found the brain-plasma ratio of CBGA methyl ester to be 0.13, which is relatively low for a CNS drug, but nearly a seven fold improvement over CBGA (Anderson et al. 2019b). Because CBGA methyl ester had significantly greater, albeit limited, brain penetration than CBGA, we hypothesized that it might be more potent in vivo. Unexpectedly, CBGA methyl ester was ineffective in reducing hyperthermia-induced seizures of *Scn1a*^+/-^ mice. This suggests that the carboxylic acid moiety may have some importance in the anticonvulsant actions of CBGA.

It is conceivable then that the methyl ester substitution disrupts the activity of CBGA at key molecular target(s) responsible for its anticonvulsant efficacy. While the anticonvulsant mechanism(s) of action for CBGA is currently unknown, pharmacological activity at epilepsy-relevant targets has been characterized with inhibition of both GPR55 and Ca_v_3.1 channels at therapeutically relevant concentrations (Anderson et al. 2021a, 2021band unpublished data). Notably, some have suggested that the GPR55 inhibition accounts for the anticonvulsant effects of CBD (Gray and Whalley 2020; Kaplan et al. 2017; Vilela et al. 2017), while Ca_v_3.1 is a target that has been associated with anticonvulsant efficacy (Calhoun et al. 2017; Chen et al. 2014; Ross et al. 2008). CBGA methyl ester, however, had no effect at GPR55 or the T-type calcium channels at 10 μM as would be predicted if these targets account for the anticonvulsant efficacy of CBGA. However, olivetolic acid had modest anticonvulsant effects in *Scn1a*^+/-^ mice but was also ineffective at these molecular targets implying the importance of other targets with this ligand. Future studies could examine the effects of olivetolic acid at alternate epilepsy-relevant targets common to CBD and CBGA such as GABA_A_ receptors, TRPV1 and voltage-gated sodium channels (Anderson et al. 2019a; 2021; Bakas et al. 2017; Ghovanloo et al. 2018; Gray and Whalley 2020; Iannotti et al. 2014). Alternatively, the anticonvulsant efficacy could be the effect of an active metabolite. Future studies could determine the metabolic profile of olivetolic acid and explore time-dependent studies to assess the anticonvulsant potential of possible metabolites.

Olivetolic acid increased the GTCS temperature threshold by approximately 0.4°C in *Scn1a*^+/-^ mice at 100 mg/kg, which is comparable to the approximately 0.5°C temperature threshold increase elicited by 100 mg/kg CBD (Anderson et al. 2019a, 2020; Kaplan et al. 2017). However, CBGA was effective in the same model at 30 mg/kg and increased the threshold temperature by nearly 1°C at 100 mg/kg (Anderson et al. 2021b). The anticonvulsant efficacy of olivetolic acid, but not CBGA methyl ester, suggests that the carboxylic acid moiety may be important to the effect. Notably, CBG is not effective against hyperthermia-induced seizures in *Scn1a*^+/-^ mice, but the phytocannabinoid acids CBCA, CBDA, CBDVA, and CBGVA are all anticonvulsant (Anderson et al. 2019b, 2021a, 2021b). The unstable carboxylic acid moiety, however, presents a challenge for the development of these phytocannabinoids as mainstream pharmaceuticals. Future studies could examine other bioisosteric replacement strategies to improve physicochemical properties, brain uptake, and stability of the anticonvulsant phytocannabinoid acids (Ballatore et al. 2013).

## Conclusion

Here we show that the cannabis precursor molecule olivetolic acid displayed some anticonvulsant activity against hyperthermia-induced seizures in the *Scn1a*^+/-^ mouse model of Dravet syndrome. However, the structurally related CBGA methyl ester compound was ineffective. Future studies could examine whether the anticonvulsant activity of olivetolic acid extends to other animal models of epilepsy.

## Supplementary Information

**Additional file 1.**

## Data Availability

The datasets used and/or analyzed during the current study are available from the corresponding author on reasonable request.
