# Supplementary material for: Olivetolic acid, a cannabinoid precursor in Cannabis sativa, but not CBGA methyl ester exhibits a modest anticonvulsant effect in a mouse model of Dravet syndrome
Source: J Cannabis Res. 2022 Jan 4;4:2. doi: 10.1186/s42238-021-00113-w (PMC8725448; doi:10.1186/s42238-021-00113-w)
Supplement: Supplementary file 1 — Additional file 1. [file 42238_2021_113_MOESM1_ESM.pdf]

Supplementary Information for

Olivetolic acid, a cannabinoid precursor in *Cannabis sativa,* but not CBGA methyl ester exhibits a modest anticonvulsant effect in a mouse model of Dravet syndrome

Lyndsey L. Anderson, Michael Udoh, Declan Everett-Morgan, Marika Heblinski, Iain. S. McGregor, Samuel D. Banister and Jonathon C. Arnold

Jonathon C. Arnold
Email: jonathon.arnold@sydney.edu.au

This PDF file includes:

Supplemental Figures 1 and 2

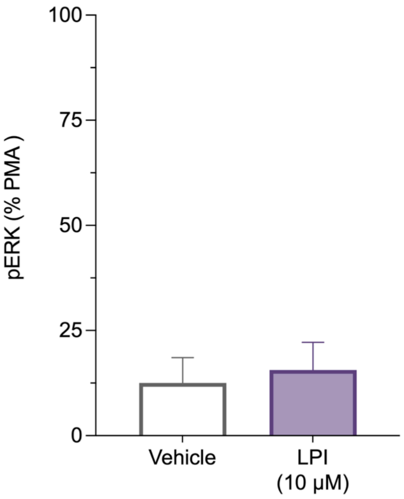

Supplemental Figure 1. GPR55 negative control experiments. Control HEK293 cells were used to measure ERK phosphorylation (pERK) following treatment with vehicle or 10 µM lysophosphatidylinositol (LPI). Data are expressed as mean ± SEM normalized to 100 nM phorbol 12-myrisate 13-acetate (PMA), with n = 5 per group.

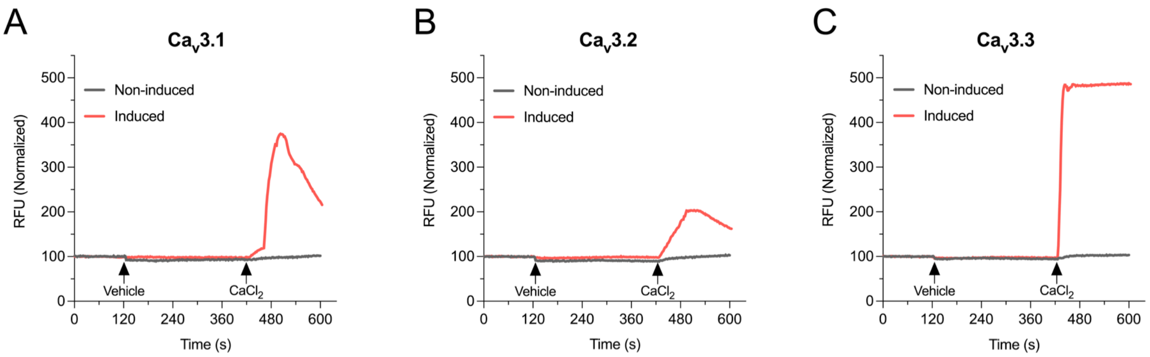

Supplemental Figure 2. Representative traces of changes in intracellular calcium. Representative traces recorded from Flp-In™ T-REx™ HEK stably expressing either (**A**) $Ca_V3.1$, (**B**) $Ca_V3.2$ or (**C**) $Ca_V3.3$ were incubated overnight with (induced, red lines) or without (non-induced, gray lines) 2 µg/mL tetracycline. Vehicle was added at 120 s followed by the addition of 10 mM CaCl2 at 420 s. Data are expressed in relative fluorescence units (RFU) normalized as a percentage to the fluorescence pre-vehicle.
